# Mobile Apps in Cardiology: Review

**DOI:** 10.2196/mhealth.2737

**Published:** 2013-07-24

**Authors:** Borja Martínez-Pérez, Isabel de la Torre-Díez, Miguel López-Coronado, Jesús Herreros-González

**Affiliations:** ^1^University of ValladolidDepartment of Signal Theory and Communications, and Telematics EngineeringUniversity of ValladolidValladolidSpain; ^2^Institute of Biomedical Engineering and Health Technology2IByTSCatholic University of MurciaMurciaSpain

**Keywords:** apps, cardiology, heart, m-health, mobile applications

## Abstract

**Background:**

Cardiovascular diseases are the deadliest diseases worldwide, with 17.3 million deaths in 2008 alone. Among them, heart-related deaths are of the utmost relevance; a fact easily proven by the 7.25 million deaths caused by ischemic heart disease alone in that year. The latest advances in smartphones and mHealth have been used in the creation of thousands of medical apps related to cardiology, which can help to reduce these mortality rates.

**Objective:**

The aim of this paper is to study the literature on mobile systems and applications currently available, as well as the existing apps related to cardiology from the leading app stores and to then classify the results to see what is available and what is missing, focusing particularly on commercial apps.

**Methods:**

Two reviews have been developed. One is a literature review of mobile systems and applications, retrieved from several databases and systems such as Scopus, PubMed, IEEE Xplore, and Web of Knowledge. The other is a review of mobile apps in the leading app stores, Google play for Android and Apple’s App Store for iOS.

**Results:**

Search queries up to May 2013 located 406 papers and 710 apps related to cardiology and heart disease. The most researched section in the literature associated with cardiology is related to mobile heart (and vital signs) monitoring systems and the methods involved in the classification of heart signs in order to detect abnormal functions. Other systems with a significant number of papers are mobile cardiac rehabilitation systems, blood pressure measurement, and systems for the detection of heart failure. The majority of apps for cardiology are heart monitors and medical calculators. Other categories with a high number of apps are those for ECG education and interpretation, cardiology news and journals, blood pressure tracking, heart rate monitoring using an external device, and CPR instruction. There are very few guides on cardiac rehabilitation and apps for the management of the cardiac condition, and there were no apps that assist people who have undergone a heart transplant.

**Conclusions:**

The distribution of work in the field of cardiology apps is considerably disproportionate. Whereas some systems have significant research and apps are available, other important systems lack such research and lack apps, even though the contribution they could provide is significant.

## Introduction

According to reports from the World Health Organization, an estimated 51 million people died in 2008 from any type of disease, communicable and noncommunicable, with the latter causing the most deaths. Among them, the worldwide leaders in mortality are cardiovascular diseases (CVDs), with 17.3 million deaths in 2008 alone, representing 30% of all deaths globally. Moreover, these deaths occur in a disproportionate way, with more than 80% occurring in low- and middle-income countries. Regrettably, no better outlook is expected, since it is estimated that more than 23 million people will die annually from CVDs by 2030 [1-5].

Ischemic heart disease (or coronary heart disease) is especially fatal among CVDs, responsible for 7.25 million deaths in 2008. Further, there are more heart-related diseases with a significant fraction of deaths, such as hypertensive heart disease and inflammatory heart disease. In fact, more people die of heart malfunction than of AIDS and all cancers combined. The contribution of these diseases to disabilities is notable—62,587 million Disability-Adjusted Life Years (DALYs) are due to coronary heart disease. These data mean massive costs to the economy of all countries, being estimated at US$448.5 billion in the United States alone in 2008 [2,6-8].

In light of such statistics, it is imperative that we reduce these numbers, not only in health care environments such as hospitals or primary health care facilities, but also in patients’ homes and workplaces. To meet this objective, mHealth, defined as “the use of mobile computing and communication technologies in health care and public health” [9], is of utmost importance. For that same reason, the latest advances in mHealth [10-13] and wireless technologies [14-16] have been used, resulting in improvements in several different aspects, from health to financial [17,18].

To aid in this initiative, it is important to acknowledge the use of smartphones and tablets as devices that have become essential to users in recent years. In numbers, there were more than 6 billion mobile subscriptions and more than 1.7 billion mobile phones sold in 2012 alone, of which 712.6 million were smartphones [19-21]. The International Data Corporation (IDC) estimated 70.9 million shipments of tablets globally in 2011 and predicted 117.1 and 165.9 million in 2012 and 2013 respectively [22]. With such growth, it was only a matter of time before the use of these devices would be adopted for mHealth, in the form of mobile applications or apps. Focusing only on the most important app stores, in terms of the market share of smartphone operating systems [23,24], the App Store [25] for Apple iOS has close to 20,000 apps in the category of Health & Fitness and more than 14,000 in Medicine. Android’s Google play [26] has more than 11,000 apps in the Health & Fitness section and roughly 5000 in the Medical apps section [27].

The aim of this paper is to conclude research on mobile applications for the most important diseases and health conditions, which we began previously with a study about mobile applications for the most prevalent health conditions [28]. This time we focus on the deadliest illness: heart disease. The main objective of this paper is to study the literature on existing mobile systems and applications, as well as cardiology apps currently available on the cited app stores. In the first part of the research, a review of published articles was performed across several systems and databases and was complemented in the second part with a search of apps in the Apple and Google app stores. The objective was to classify the results in order to see the progress as well as the lack of applications and systems, focusing specifically on commercial apps (ie, those apps available on app stores). Other secondary objectives were to obtain information regarding the prices of these apps and their target users. Since there are no published reviews on this specific topic, the results may be of considerable interest for developers and researchers wanting to investigate further or to create a new app.

## Methods

Two reviews were developed and took place up to May 2013. The first was a review of existing mobile systems and applications in the field of cardiology found in the literature, and the second, a study of commercial apps related to cardiology found in the leading app stores.

### Review of Mobile Systems and Applications for Cardiology in Literature

**Figure 1:**
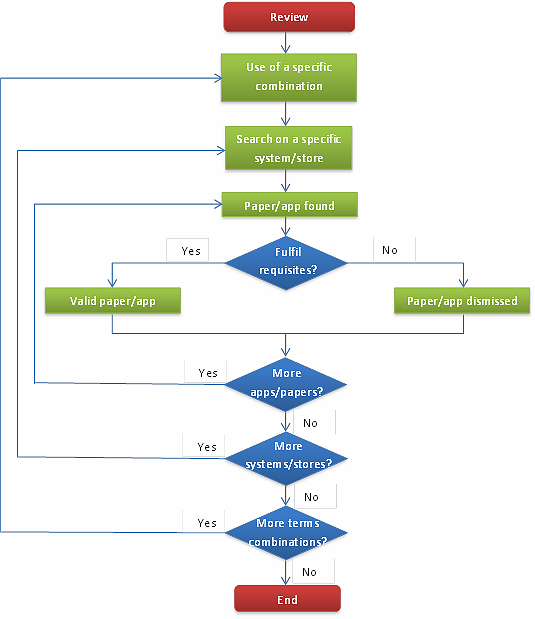
Flow chart of the methods followed in the literature and the commercial reviews.

To perform the literature review, the following systems and databases were used: Scopus, IEEE Xplore, Web of Knowledge, and PubMed. The methodology followed is presented in Figure 1 and also applies to the commercial apps review. In this section, the objective was to search for relevant papers in different systems. The following combinations of search terms were used in the metadata field: “term1” AND mobile AND (application OR app); “term2” AND mhealth; “term2” AND smartphone; and “term2” AND “mobile phone”, where “term1” and “term2” could be different words. The words used for “term1” were heart disease, heart failure, infarction, arrhythmia, heart attack, coronary disease, angina, fibrillation, cardiology, hypertension, heart transplant, and heart transplantation. For “term2”, the terms used were cardiology and heart.

The results were limited to the last 10 years, from 2003 to the present day, and the eligibility requisites used were the following: only papers published originally in English were studied. Publications about mobile systems or applications not focused on cardiology or designed for more than 3 different fields were dismissed, but if the application was intended for 3 disciplines or fewer with being cardiology one of them, then it was included in the review. Similarly, papers regarding systems for many diseases (not only cardiac diseases) were rejected. Articles with algorithms for classification, detection, compression, encryption, or authentication of ECG data (or other types of heart signs) were studied, but publications about the possible influence of the electromagnetic fields irradiated by mobile phones in the heart rate or in implanted cardiac devices were dismissed.

When the selection of papers was finished, the authors convened in order to classify the articles in different categories by reading their abstracts as well as the whole article when required. Once this process was completed, a revision was done and similar categories were merged to obtain a more condensed classification.

### Review of Apps for Cardiology in Commercial App Stores

The second review was a search of apps related to cardiology in the two leading commercial stores for smartphones [23,24]: the Apple App Store and Google play.

The methodology used in this review is the same used in the previous one, shown in Figure 1, although in this case exploring apps in commercial stores, instead of papers in databases. The search terms used for both stores were heart and cardiology. The eligibility requisites used for this review were the following: apps not in English or in the language of the place where the search was executed (Spanish) were dismissed, the same as those with their summary in a different language from the two mentioned. Only applications focused on cardiac issues were studied. Games, music, apps for cholesterol management or losing weight, apps for animals, and apps for congresses or conferences were dismissed, but apps related to hypertension were included in the study.

In the App Store, since the apps are separated for iPod and iPhone and for iPad, only the first ones were searched, although in many cases they were also available for iPad. During the search on Google play, some problems arose. When searching by “cardiology” or “heart”, the store indicated that there were at least 1000 results, although it showed only 480. Google was asked about this discrepancy and the issue is still under investigation; hence, it was decided to use other search words in order to obtain the highest number of apps related to cardiology in this store. These words were infarction, heart attack, heart failure, heart disease, fibrillation, coronary heart, angina, and arrhythmia. Additionally, in some of these new searches on Google play, it was indicated that a certain number of results has been found but, when exploring the pages of the results found, the last pages (usually between one and three) were blank. This issue did not affect the review since the apps studied are the ones shown, although it is not known if there were more apps that were not visible due to this error.

Once the selection of cardiology-related apps was complete, the authors convened again to sort the apps by their purpose in different types, by reading the summary and explanation given by the stores and downloading them when the explanation specified was not clear enough. In these cases, the smartphones used were an iPhone 4 if the app was designed for iOS and a Samsung Galaxy S SCL GT-I9003 in the case of an app for Android. Finally, revisions were done in order to shorten the classification, similar to the process followed in the literature review.

## Results

### Mobile Systems and Applications for Cardiology in Literature

A total of 406 relevant papers were found in all the databases and systems used. The classification of the papers by their content is shown in Table 1, which also shows the number of articles for each category.

**Figure 2:**
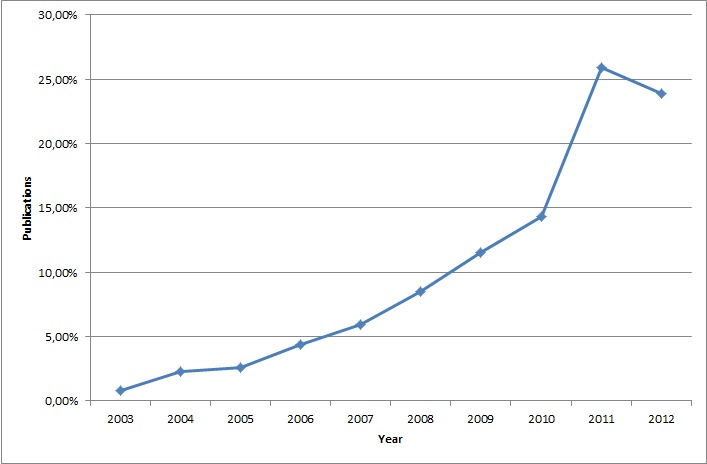
Percentage of papers found per year of publication.

In Figure 2, the percentage of papers found by their year of publication, from 2003 to 2012, is shown. It can be seen that the publications increased every year until 2011, decreasing slightly in 2012.

### Apps for Cardiology in Commercial Stores

A total of 710 relevant apps were found in the App Store and Google play, although we note that some apps are available in both stores. In this study, these apps are counted separately. Hence, 439 apps are available for iOS and 271 for Android. The majority of the apps for iOS are found in the categories of Medicine (303) and Health & Fitness (111), whereas the remaining are included in Utilities (8), Education (7), Entertaining (3), Lifestyle (2), Sports (2), Books (1), Reference (1) and Social network (1). The apps for Android are found in the categories of Medical apps (188), Health & Fitness (65), Education (8), Lifestyle (7), and Books & Reference (3). The classification of these apps sorted by their functions and in decreasing order are shown in Table 2.

**Figure 3:**
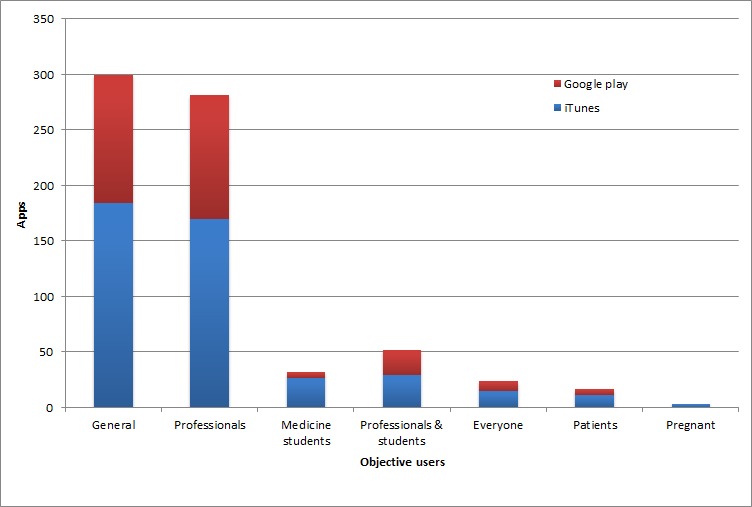
Classification of the apps of the commercial review by target users.

If the classification was performed taking into account the target public to whom the app is destined, another sort was conducted. Figure 3 shows the number of apps sorted by target users for each app store.

To understand Figure 3, it is necessary to explain the difference between the “general public” and “everyone”. General public is used for common users—normal people not necessarily affected by heart disease. It does not include health care professionals. On the other hand, everyone includes all groups. It is used for apps intended for a specific type of user, such as medical students but that can also be used by other users, such as professionals or the average person for example.

Finally, statistical values on the prices of the apps separated by commercial store are summarized in Table 3. For the mean, mode, and median values, the study is divided into two groups: one group shows all the apps found and the other shows only the apps that are not free.

**Table 1:** Classification of the results of the literature review.

Type of system/application	Articles
Vital signs monitoring system	55
ECG (electrocardiography)/cardiac signal detection/classification algorithms	44
Heart monitoring system	37
ECG/heart monitoring system trial/evaluation	28
Remote heart monitoring system	25
Heart monitoring system with alerts	17
Cardiac rehabilitation mobile system	16
ECG data transmission	14
Blood pressure measurement/monitor system	13
Remote management/monitoring of implanted pacemakers/cardiac devices	11
Teleconsultation system	10
Remote and local heart monitoring system	8
Exercising/sports related heart monitoring system	7
Innovative heart rate monitor	7
Arrhythmia detection system	7
Sensors evaluation/state of the art	6
Surveys/states of the art of cardiology systems	5
Heart rate & blood pressure monitoring system	5
Remote heart monitoring system with alerts	5
Atrial fibrillation detection system	5
Heart failure detection system	5
Phonocardiography mobile system	5
Remote and local heart monitoring system with alerts	4
Breath monitoring system	4
ECG data compression technique	4
Automatic music selector to maintain a target heart rate	4
Alerts and location of heart attacks	4
CPR instructions through mobile phone trial	4
CPR instructions/reminder through mobile phone	4
Applications for promotion of healthy behaviors	4
System for measuring/reducing stress	3
Personal lifestyle and health management system	3
Fetal heart monitoring system	3
ECG data encryption/authentication/privacy	3
Cardiac rehabilitation mobile system trial	3
Exercising/sports related heart monitoring system evaluation	3
Mobile medical applications for chronic diseases	2
Pocket-size images interpretation	2
First aid/resuscitation app evaluation	2
Apps for hypertension in smartphones	2
Emotional states detection through measuring heart rate differences	2
Weight control in high-risk heart failure population	2
Medications management	2
Telemetry-based system for monitoring rats’ vital signs	1
Heart attack self-test app	1
Trial for comparing follow-up of hypertensive patients	1
Study about the correlation music-heart rate variability	1
App for improving basic life support (BLS)	1
Non-invasive tissue classifier	1
Share of vital signs in a social network	1

**Table 2:** Classification of apps in the commercial review by function.

Type of app	Number
Heart rate monitor	94
Algorithm/calculator/predictor	85
Informative guide	41
Educational ECG/interpretation aid of ECG	37
News/journal	34
Blood pressure tracker	30
External devices heart rate monitor	22
CPR (cardiopulmonary resuscitation) instructions	21
Educational anatomy	17
Medicine students education	17
Guide/book	17
Health tips	17
Diagnosis & treatment guidelines	14
Echocardiography reference	14
Professionals & students education	14
Medicine exam preparation	13
General education	11
Diagnosis aid	10
Animated guide	9
Heart sounds reference	9
Blood pressure & heart rate monitor	9
Cardiology medical reference	8
Medical images reference	8
Catheter reference	8
Guide of professional commercial devices	7
Stethoscope/educational stethoscope	7
AED (automated external defibrillator) location	6
Professionals education	6
Clinical trials	6
ECG cases reference	5
Hypertension reference/guidelines	5
Procedures in emergency cases	5
Patients’ history/ECGs/images	4
Surgeon aid/training	4
Professionals connections, knowledge/cases share	4
Angiography reference/guide	4
Auscultation reference	4
Heart rate monitor for exercising	4
Fetal heart rate monitor/interpretation	4
Heart rate calculator	4
Ultrasound video reference	4
Resuscitation instructions/guide	4
Educational/explanations for patients	3
ECG sending	3
Log procedures	3
Diseases prevention guide	3
Heart rate monitor with external devices for exercising	3
Professionals guidelines	3
Audio reference	2
ECG signal transformer	2
Location of cardiac emergencies	2
Blood pressure measurement with external devices	2
CPR and AED instructions	2
Prescribing drugs	2
AED training	2
Pulse measurement aid	2
Hospitals	2
Instructions/training CPR & AED location	2
Perfusion reference	2
Arrhythmia reference	2
Acute coronary syndrome reference	2
Stent guide/reference	2
Prosthesis guidelines	1
Teleconsultation	1
Upload ECG from a commercial monitor	1
Medications reminder	1
Treatment guide	1
Stand-alone or with external device heart rate monitor	1
Auto-diagnosis	1
Cardiac rehabilitation guide	1
Anesthesia management	1
Social network	1
Atrial fibrillation guidelines	1
AED training with simulation of external device	1
Blood pressure prevention & treatment exercises	1
Condition management	1
Congenital heart defects reference	1
Clinical examination guide	1
Blood pressure measurement	1
Driving guidelines for cardiac patients	1

**Table 4:** Statistical data on the prices of apps in the commercial review.

	Min.	Max.	% free apps	Mean		Mode		Median	
				Total	Not free	Total	Not free	Total	Not free
iTunes	0	129.99	43.28	2.89	5.10	0	0.89	0.89	1.79
Google play	0	100	49.82	3.45	6.87	0	1.04	0.71	2.17

## Discussion

### Principal Findings

In light of the results presented in the previous section, several interesting findings can be extracted. Focusing on the literature review, the majority of the 406 articles found are about systems that normally use a mobile phone or smartphone and only a small percentage concerns mobile apps exclusively. In addition, as Table 1 shows, 4 of the first 5 types of applications with more papers are directly related to heart monitoring systems (including vital signs) while the remaining one is related to algorithms for selection/classification of ECGs or other measurable heart signs. At the same time, other different types of heart monitoring systems can be found in the classification. There were 198 papers about those systems, which is almost half of all the publications found. This indicates that the most researched area associated with cardiology is related to mobile heart monitoring systems and the techniques involved in the classification of heart signs in order to detect malfunctions.

An example of a heart monitoring system is the one proposed by Yap et al (2012), which uses a chest-belt wireless ECG measurement system in combination with an Android app for monitoring ECG in real time [29]. In this case, there is an app involved in a whole system, but, as mentioned above, there are few articles focused exclusively on apps, such as the one written by Leijdekkers and Gay (2008) about a heart attack self-test app that allows possible victims to assess whether they are suffering an infarction [30]. De Vries et al (2013) evaluated the actual use and goals of telemonitoring systems [31], whereas Seto et al (2012) developed a randomized trial of mobile phone-based telemonitoring systems [32] to examine the experience of heart failure patients with these systems [33]. An algorithm for improving the classification of ECGs was developed by Jekova et al (2011), which scores the noise corruption level of ECG data by evaluating several features [34]. It is important to indicate that most of these systems cited were designed for patients’ use with the knowledge and consent of their professional caregivers, since they have an important role in the correct functioning of the applications, either monitoring remotely the patients’ heart or receiving alarms when a heart problem occurs.

Other systems with a number of papers are the following: cardiac rehabilitation mobile systems [35,36], extremely important in the recovery of heart attacks or in their prevention in people with heart problems [37,38]; systems for measuring blood pressure combined or not with heart rate monitors [39,40], in order to avoid possible problems derived from hypertension (or raised blood pressure), an important risk factor for coronary heart disease or ischemic stroke and indirectly the cause of 9.4 million deaths every year [41,42]; and systems for the detection of heart malfunctions, sometimes focused on certain problems such as arrhythmia or atrial fibrillation [43] and sometimes considering more than one disease [44]. Other important contributions are done in the remote management or monitoring of cardiac devices such as pacemakers, in order to assess their correct operation and to perform periodic checks, which is currently very common in modern health care facilities [45].

Examining the dates of publication of these papers, shown in Figure 2, it is clear that research in mobile systems in the field of cardiology has gained more and more importance in recent years, beginning in 2003 and with many more published articles by 2011 and 2012. This fact shows that the part of cardiology associated with mobile technology has become the recent focus of investigations—quite logically since cardiovascular diseases and especially heart diseases are the leading causes of death worldwide.

**Figure 4:**
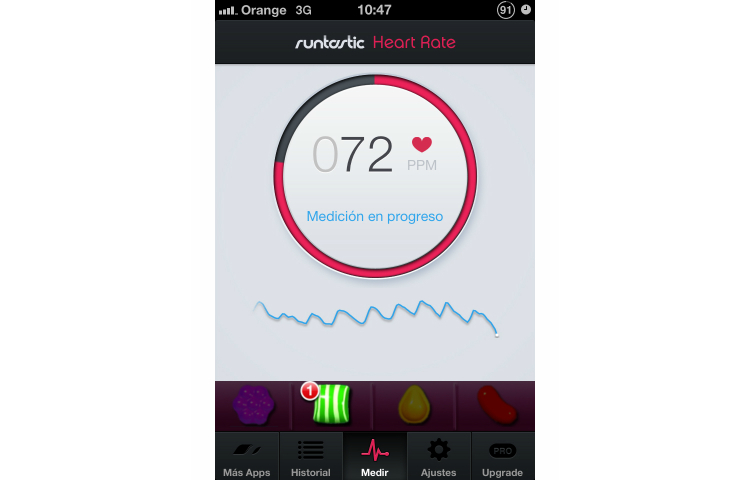
Snapshot of Runtastic Heart Rate.

**Figure 5:**
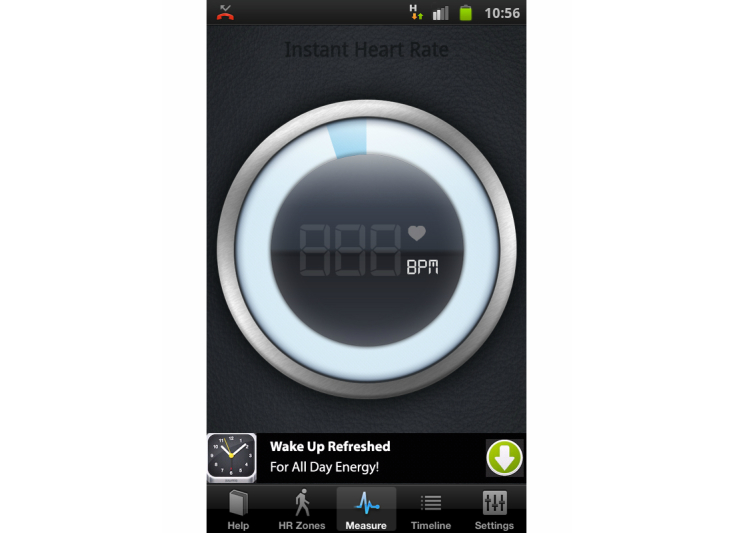
Snapshot of Instant Heart Rate.

In the review of cardiology-related apps, many outcomes were observed. The first notable conclusion from classification (Table 2) is the difference between the first two positions (ie, heart rate monitors and calculators) and the rest, when taking into account the number of apps for each category. There are far more apps for heart rate monitoring and medical calculators or predictors than for informative guides, which is in the third position. In addition to these heart monitoring apps, there are those that use an external device as well as those focused on exercising. Figures 4 and 5 show snapshots of two examples of heart monitoring apps: Runtastic Heart Rate & Pulse Monitor [46] for iOS and Instant Heart Rate [47] for Android. Of the more than 20 apps, we found the following types: apps for ECG education and interpretation, cardiology news and journals, blood pressure tracking, heart rate monitoring using an external device, and apps with instructions about CPR. These categories equal a total of 364 apps, which is more than half of the total apps found.

Despite the fact that the classification shown in Tables 2 and 3 is subjective, we believe we obtained the most realistic classification, performing several iterations until arriving at the final one. As a result, those categories with similar patterns and characteristics have been merged, whereas others with unique features were not, in order not to lose information in the process. As a result, there are many categories with few apps, some of them highly useful and interesting, such as apps related to resuscitation procedures, including apps with CPR and/or AED instructions, essential for properly performing a resuscitation and, hence, saving lives. There were also AED location apps, which indicate the position of nearby AEDs for cardiac emergencies with the help of the GPS (Global Positioning System) included in every modern smartphone. Other compelling apps are those designed to locate cardiac emergencies, through the help of the users, either sending an emergency alarm or receiving it in order to assist them on site. The majority of these types of apps are free; therefore, their use has no additional cost to the user. Since the outcomes they can achieve are significant (eg, saving the life of a person suffering a heart attack), it would be better if there were more available and they were also free.

There are other categories with very few apps (or even no apps), which could provide significant assistance and help. Examples are guides for cardiac rehabilitation and apps for the management of a cardiac condition, with only one app of each type.

Since a rehabilitation program for people who have suffered or suffer heart problems (or have had a heart surgery) is vital [37,38] in order to avoid more worrisome consequences, it would be beneficial for these individuals to have a guide with exercises and instructions for complete rehabilitation of their hearts. Similarly, apps for the management of heart conditions can be useful for people suffering congenital heart defects involving arrhythmia, angina, fibrillations, etc. In addition to this, it is surprising that there are no apps for aiding people who have undergone a heart transplant, since guidelines, exercises, or even medication management would be of help.

Figure 3 shows the numbers of apps for a specific target public, where it is clear that the users preferred by developers are general users and health care professionals. Typical apps for the general public are heart rate monitors, blood pressure trackers, apps with health tips, educational apps, and resuscitation (CPR and AED) guides. Common apps for professionals are calculators and predictors useful for diagnosis or treatment, specific educational apps and references (angiography, catheters, surgery, etc), and guides, books, and apps for assisting in the diagnosis. Educational apps about medicine and apps for the preparation of medical exams are usually designed for students. The apps intended for common people, medical students, and professionals (category “Everyone”) are usually educational and informative apps while typical apps for patients are those for the care of their condition, some with calculators to assess their state and informative guides. The apps for pregnant women are fetal heart rate monitors. Here we note that there are few apps designed exclusively for patients. Nevertheless, there are many general informative applications that can be used by patients; hence, these apps can compensate for the lack of specific apps for patients. However, a preferable option would be the creation of a specific app for the treatment of a determined condition in order to help the affected in a more appropriate way.

### Conclusions

Comparing the results for each store, several conclusions can be made. First, there are more apps related to cardiology in the App Store, despite the fact that Android is more prevalent than iOS [23,24]. Figure 3 shows that the numbers related to target users obtained for Apple are consistent with the numbers for Google play, suggesting that the developers for both stores have similar approaches and ideas. The data shown in Table 3 reveal interesting findings. Google play exceeds the App Store only in the number of free apps. Despite the fact that the highest-price app on the App Store is more expensive than the corresponding on Google play, the mean price is lower than the one in Google play, whether free apps are included or not. This was unexpected, since there is a collective belief that iOS devices (especially iPhones and iPads) are more exclusive (usually used in the business environment) and expensive than Android devices [48,49], and their apps are also considered to be higher quality than apps for Android [27,50]. This seems not to be the case in the cardiology field.

There are various lines of investigation for further research. New applications are needed for the management and monitoring of specific cardiac conditions, designed for the patients affected by them, since there are few currently available. The development of applications with this aim could be rather valuable. Another potential field of research could be the creation of an app with guidelines and information for people who have undergone a heart transplant. Such an app could assist them in their new condition and facilitate their new state. Another field to supply is related to resuscitation guides and instructions. Although there are several useful apps with such functions, the creation of an app with different functionalities parallel to the existing ones could be another beneficial field of research, perhaps developing the idea of the location of cardiac emergencies for people trained in resuscitation skills.

## Abbreviations

AED: automated external defibrillator
BLS: basic life support
CPR: cardiopulmonary resuscitation
CVDs: cardiovascular diseases
DALYs: disability-adjusted life years
ECG: electrocardiography
GPS: Global Positioning System
IDC: International Data Corporation
WHO: World Health Organization
